# The Effects of Ketogenic Diet on Insulin Sensitivity and Weight Loss, Which Came First: The Chicken or the Egg?

**DOI:** 10.3390/nu15143120

**Published:** 2023-07-12

**Authors:** Antonio Paoli, Antonino Bianco, Tatiana Moro, Joao Felipe Mota, Christianne F. Coelho-Ravagnani

**Affiliations:** 1 Department of Biomedical Sciences, University of Padua, 35127 Padua, Italy; tatiana.moro@unipd.it; 2 Research Center for High Performance Sport, UCAM, Catholic University of Murcia, 30107 Murcia, Spain; 3 Sport and Exercise Sciences Research Unit, University of Palermo, 90144 Palermo, Italy; antonino.bianco@unipa.it; 4 School of Nutrition, Federal University of Goiás, Goiânia 74605-080, Brazil; jfemota@gmail.com; 5 APC Microbiome Ireland, Department of Medicine, School of Microbiology, University College Cork, T12 YT20 Cork, Ireland; 6 Research in Exercise and Nutrition in Health and Sports Performance-PENSARE, Post-Graduate Program in Movement Sciences, Institute of Health (INISA), Federal University of Mato Grosso do Sul, Campo Grande 79070-900, Brazil; christianne.coelho@ufms.br

**Keywords:** ketogenic diet, insulin sensitivity, insulin resistance, weight loss, body weight, very low carbohydrate diet

## Abstract

The ketogenic diet (KD) is, nowadays, considered an interesting nutritional approach for weight loss and improvement in insulin resistance. Nevertheless, most of the studies available in the literature do not allow a clear distinction between its effects on insulin sensitivity per se, and the effects of weight loss induced by KDs on insulin sensitivity. In this review, we discuss the scientific evidence on the direct and weight loss mediated effects of KDs on glycemic status in humans, describing the KD’s biochemical background and the underlying mechanisms.

## 1. Introduction

In the last decades, the world faced a pandemic increase in overweight and obesity [1]. The World Health Organization (WHO) estimates that 39% and 13% of world’s adult population are overweight and obese, respectively. Alarmingly, the prevalence of overweight among children and adolescents escalated from 4% in 1975 to 18% in 2016 [1]. Overweight and obesity are related to insulin resistance (IR), defined as a defect in the insulin-mediated control of glucose metabolism, predominantly in muscle, fat and liver tissues [2]. In response to such condition, there is an excess of insulin secretion in an attempt to normalize blood glucose concentration [3]. IR represents a pre stage for type two diabetes, and shows a substantial increase in conjunction with other related diseases such as non-alcoholic fatty liver disease (NALFD) [4,5].

The complex etiopathogenesis of this dysmetabolic pandemic involves a dangerous mix of physical inactivity and unhealthy dietary patterns. Regarding physical inactivity, it has been reported that about one third of adults worldwide fail to achieve the recommended activity levels [6]; therefore, about 7 to 8% of all-cause and cardiovascular disease deaths are attributable to physical inactivity [7]. This substantial non-communicable disease burden of physical inactivity is a common feature for high, middle, and low-income countries [7] and, together with unhealthy diet [8], may also explain the increase in IR all around the world. The epidemiology of isolated IR (not associated with other criteria necessary to define the metabolic syndrome) shows different distribution among countries, with prevalence varying from 44.8% in a cohort of young adults in US [9], 46.5% in Venezuelan adults [10], to 15.5% to 48.11% in Europe, depending on which population was analyzed [11,12,13].

One of the more promising nutritional approaches to improve IR is the ketogenic diet (KD). The KD is a diet in which the amount of carbohydrate is limited to less than 50 g per day, whilst the intake of fat is increased to assure an adequate energy intake [14]. Indeed, many studies have shown a favorable effects of KD on insulin resistance in subjects with overweight or obesity [15,16,17,18,19]; however, some studies observed, intriguingly, a significant improvement in insulin sensitivity in response to low carbohydrate diets even in the absence of weight loss [20,21,22]. Therefore, our goal is to review the scientific evidence on the dependent and weight-loss-mediated effects of the KD on glycemic status, describing the biochemical background of the KD and the underlying mechanisms. As animals may differ from humans in their responses to diet (considering also the different lifespan and its ratio to diet duration, 14 months of KDs in mice correspond to 48 years in a human being [23]), we have drawn the effects of KDs on insulin sensitivity outcomes only from human studies.

## 2. Insulin Resistance

### 2.1. Pathophysiology of Insulin Resistance

The insulin action is guaranteed by a cellular signaling cascade, involving membrane insulin receptors (IRS) and intracellular proteins (PI3K and AKT) [24]. These protein-protein interactions are essential to plasma glucose uptake into tissues. By contrast, impairments in cellular signal transduction and in insulin actions in response to insulin stimulation—labeled as IR [24]—compromise glucose control leading to the development of Type 2 diabetes (T2DM).

There are three main tissues involved in IR: muscle, liver, and adipose tissue. It is well-known that IR is related to overweight/obesity tissues’ exposure to high energy density foods and consequently accumulation of related toxic by-products (such as reactive oxygen species (ROS) and ceramides) together with immune-mediated inflammatory events, hormonal changes, and activation of intracellular stress response pathways [3,25,26].

#### 2.1.1. Skeletal Muscle

Skeletal muscle is the main tissue responsible target for glucose disposal (about 70%) after a caloric load. However, when the needs for skeletal muscle glucose uptake exceed its capacity (called metabolic overload), the excess glucose moves to the liver causing de novo lipogenesis (DNL) [27,28,29]. The increased flux of fatty acids and the metabolic overload in muscle cause an increase in intramuscular diacylglycerols (DAGs), long-chain acyl CoAs (LC-CoAs), ceramides, and triglycerides (TG). The intramuscular levels of LC-COAs, DAGs and ceramides are positively correlated to muscle TG content and negatively with insulin sensitivity [30]. The exact pathway of this causal relationship is still under debate; although some data, analyzing the IR induced by palmitate (but not by linoleic acid) through an increase in ceramide biosynthesis, suggest a central role of the inhibition of the protein kinase B/Akt pathway mediated by ceramides [31]. Moreover, the transgenic overexpression of diacylglycerol acyltransferase 1 (DGAT1) (that catalyzes the final step in the Kennedy pathway for TG biosynthesis) in muscle, increases the skeletal muscle TG content, decreases its DAGs and ceramide levels and prevents IR induced by the diet [32]. The metabolic overload and the excess of dietary fats stimulate the activation of target genes promoting B-oxidation without a contextual increase in tri-carboxylic acid cycle (TCA) flux [25]; this leads to a overproduction of metabolic by-products (ROS, acylcarnitine) related to the incomplete oxidation. The by-products then accumulate in mitochondria [33,34] activating Ser kinases that interfere with insulin signaling and GLUT4 translocation [33].

#### 2.1.2. Liver

The same mechanism described for skeletal muscle works for the liver; the metabolic overload and the increased lipids flux increase the concentration of DAGs, LC-CoAs, ceramides, and TG mainly through the increased level of malonyl CoA, which serves as precursor for DNL but also as the inhibitor of carnitine palmitoyl transferase-1 (CPT1) which is fundamental for the entry of LC-COAs into the mitochondria for the B-oxidation [35]. Moreover, the inhibition of the expression of B-oxidative enzymes in the liver induced by the effect of insulin on PGC1α (peroxisome proliferator-activated receptor-γ (PPARγ) co-activator-1α) facilitates the increase in ectopic fat in the liver leading to IR [36].

#### 2.1.3. Adipose Tissue

A normal quality/quantity of white adipose tissue is fundamental for the normal regulation of insulin action, as demonstrated by animals lacking adipose tissue which exhibit severe hepatic and muscle IR with a contextual increase in TG concentration in the tissues [37,38]. On the other hand, the impaired antilipolytic effect of insulin on adipose tissue increases the efflux of circulating free fatty acids (FFAs) that, in turn, act through the above mechanisms on skeletal muscle and liver [39]. Moreover, the increase in adipose tissue (mainly visceral adipose tissue (VAT)) *di per se* creates an inflammatory milieu that worsens local, liver and muscle insulin sensitivity [40]; this scenario, together with the higher rate of FFAs mobilized from the adipocytes of VAT [41], explain the vicious circle of obesity and IR [42].

### 2.2. Metabolic Inflexibility

Insulin resistance is also associated with an impairment in both oxidative and non-oxidative glucose metabolism. Indeed, the ability of skeletal muscle to switch between lipid and glucose oxidation, defined as metabolic flexibility, is impaired in IR individuals with obesity and in patients with T2DM [43]. Metabolic flexibility can be estimated by calculating the difference between the respiratory quotient (RQ) in the basal state and during insulin stimulation [44], diet challenges [45] or exercise [46]. In healthy lean individuals, the RQ is around 0.83 in the normal morning fasting state and may rise to over 1.0 in the postprandial or insulin-stimulated state, reflecting the switch from a predominant lipid to glucose oxidation, whilst after many hours of fasting (>24) or after few days of ketogenic diet (which will be discussed later) can drop to 0.7. Subjects with IR obesity and T2DM who exhibit metabolic inflexibility have elevated fasting RQ [47]. This reduced capacity of some tissues to adjust lipid oxidation leads to lipid accumulation in tissues and lipotoxicity which, in turn, impair insulin signaling [47]. The question whether the reduction in metabolic flexibility in patients with obesity and/or T2DM is a consequence of IR or represents a pre-condition is still under debate [48].

However, considering the pivotal role of excess adipose tissue in IR pathophysiology, a reduction in body weight may lead to an improvement in insulin sensitivity [49,50,51]. However, “weight loss is not a prerequisite for improvement in insulin sensitivity” [11] since it could be achieved through the direct effects of exercise (outside the aim of this review) [52] or through different dietary manipulation [53].

## 3. Direct Effects of Weight Loss on Insulin Sensitivity

Although not all individuals with obesity are insulin resistant, there is a well-demonstrated connection between these two phenomena, which is largely attributed to adipose and others tissues’ metabolic dysfunctions [54]. The hormonal milieu facilitating the IR within the adipose tissue is worthy of more substantial discussion. Indeed, adipocytes secrete several hormones (adipokines) that can act on glucose and lipid metabolism as well as both anti-inflammatory or inflammatory mediators [55]. Adiponectin is an anti-inflammatory adipokine with well known insulin-sensitizing effects, acting through tissue receptors (adipoR1, adipoR2 and T-cadherin) causing an activation of AMP kinase and PPAR, and a decrease in circulating lipid levels, hepatic glucose output and fatty acid synthesis along with an increased muscle glucose uptake and fatty acid oxidation [56,57,58]. Adiponectin levels are reduced in obesity and T2DM [57] and increased during weight loss [59,60]. The negative feedback on adiponectin production explains the hypoadiponectinemia frequently recorded in subjects with obesity (i.e., the expression of adiponectin is activated during adipogenesis, but its production is inhibited in the development of obesity) [61]. Furthermore, insulin sensitivity is a requirement for adiponectin secretion. Thus, subjects with obesity who are more prone to have mild systemic inflammation and insulin resistance would be at higher risk of hypoadiponectinemia [62]. In this context, the elevation of plasma adiponectin induced by weight loss could suggest an induction of its production as well as an improvement in the insulinemic and inflammatory profile [61,62]. The higher omental expression of adiponectin by the smaller adipocytes could be another good explanation behind increased adiponectin levels induced by weight loss interventions [63]. Taken together, these mechanisms underscore the key role of weight loss (especially fat loss) in adiponectin-mediated insulin sensitivity. Importantly, other adipokines involved in insulin action have been described, such as resistin, retinol-binding protein 4, and omentin [55]. However, it remains unclear how and to what extent these proteins are affected by weight loss.

As aforementioned, changes in adipose tissue quality/morphology (not only quantity) are another important feature of weight loss and glucose control. A fat excess, related to enlarged adipocytes is negatively associated with insulin sensitivity [49,55,64] more than the one related to increased numbers of adipocytes. In this regard, a prospective study with women with obesity undergoing bariatric surgery showed a marked fat mass reduction (50%) associated with a decrease in abdominal subcutaneous adipocyte volume (but not number), which was significantly associated with improvements in insulin levels [64]. As expected, the decrease in visceral but not subcutaneous fat mass (assessed by DEXA) was associated with improved insulin sensitivity (probably because the liver is directly exposed to releasing factors from visceral adipocytes via the portal vein). However, the baseline analysis (before surgery) showed that subcutaneous fat cell size was more strongly correlated with insulin sensitivity than visceral fat cell volume, suggesting a greater importance of “size” than “volume” of adipocytes for insulin sensitivity. All of these results were associations and should be interpreted with caution. Further well-controlled studies should assess the direct impact of adipose tissues on insulin sensitivity.

Different forms of weight-loss approaches (i.e., surgery, low energy diets, ketogenic diets, exercise) are accompanied by a decrease in fat cell size [65] and volume [59,66] with consequent improvements in insulin sensitivity. In this regard, some mechanisms have been proposed: one is related to the greater capacity of smaller subcutaneous adipocytes to store lipids (reduced by volume as a consequence of weight loss) that can result in less ectopic lipid deposition, facilitating the insulin signaling in peripheral tissues, especially in liver, pancreas and skeletal muscle [49]. It is noteworthy that liver glucose production is determined, in part, by hepatic insulin sensitivity, which is impaired by the high content of fatty acids in hepatocytes [67]. Notably, in patients with T2DM, a reduction in gluconeogenesis and in plasma glucagon concentration (−25%) was observed even in moderate weight reduction (~8% of body weight) which were mostly associated with marked reductions in intrahepatic lipid content [68]. This and other findings [67]] reinforce the role of weight loss to attenuate or even reverse hepatic IR, largely mediated by liver fat reductions. Additionally, small adipocytes exhibit an increased secretion of anti-inflammatory adipokines, and decreased pro-inflammatory adipokine release such as tumor necrosis factor-α (TNF-α) and interleukin-6 (IL-6) [55]. The adipose tissue inflammation is likely a causal factor for widespread systemic IR in obesity [55]. Cytokines can interfere directly with insulin signaling by activating *c*-Jun *N*-terminal kinase but also by increasing basal lipolysis of adipose tissue and exacerbating ectopic lipid accumulation and lipotoxicity [69]. Thus, the amelioration of inflammatory adipokine release triggered by weight loss might be of great importance in improving whole body insulin responsiveness.

Considering the possibility of developing IR before any changes in infiltration of circulating cytokines and immune cell into adipose tissue, Morigny et al. [69] postulate the dysregulation of adipocyte basal lipolysis as a primary event contributing to IR in obesity. According to the authors, the increased basal fatty acids release from adipose tissue in subjects with obesity can:

(1) Worsen lipid ectopic accumulation (see previous paragraph);
(2) Interfere negatively with the release of adipokines;
(3) Cause inflammation and adipose tissue macrophage accumulation.

Moreover, the uptake of free fatty acids from the blood (as noted above) leads to a hepatic overproduction of very low density lipoprotein (VLDL) resulting in hypertriglyceridemia and lower HDL-c levels, which seem to be a common feature in subjects with IR obesity [70]. In this context, dyslipidemia could be a marker of IR but also a causal factor. The literature on the effects of weight loss on lipolysis is sparse and also contradictory [71], thus the precise mechanisms remain to be elucidated. However, decreases in basal lipolysis and consequent improvements in insulin sensitivity were observed in weight loss induced by surgery [72] and dietary intervention [73]. Although some of their underlying mechanisms can be slightly different, the most plausible explanations are related to a decline in abdominal fat mass, adipocyte size and hormone-sensitive lipase function, all associated with improvements in adipocyte IR after weight loss. Interestingly, after a very low calorie diet intervention, the decrease basal lipolysis in women with obesity was accompanied by an increase in B-adrenoceptor sensitivity suggesting a more efficient regulation of lipolysis as a result of weight loss [73].

Overall, in response to weight loss, adipose tissue experiences quantitative and qualitative (morphological and functional) changes that lead to improvements in insulin sensitivity. In these terms, treatments aimed at reaching health glycemic targets should primarily focus on achieving weight loss. The intriguing mechanisms by which diet enhances insulin action apart from weight reduction will be discussed in the next section.

## 4. Ketogenic Diet

As the prevalence of IR has risen in recent years, different approaches and interventions have been tested and studied toward reducing it. General approach has been to reduce the intake of ultra-processed foods which typically contain nutrient profiles that are implicated in the risk of IR: hydrogenated oil, fructose corn syrup, low amounts of fiber, and different food additives [74]. Moreover, many guidelines suggest the beneficial effects of a diet rich in whole grains and high amounts of non-starchy vegetables and raw fruits [75].

Some studies have observed, intriguingly, a significant improvement in insulin sensitivity in response to low carbohydrate diets even in the absence of weight loss [20,21,22]. Considering that, the dietary patterns are defined by the cultural backgrounds, personal preferences, clinical conditions, and socio-demographic and economic settings in which the individuals live, and therefore, one diet “does not fit all”, it would be of interest to discuss the role of KDs on IR.

The KD was first proposed by Dr. Wilder in 1921 as as a more feasible alternative nutritional therapy to fasting for epilepsy [76]. Five years later, Dr. Peterman at the Mayo Clinic proposed the first KD protocol of 1 g/protein/kg/day, 10 to 15 g carbohydrates/day, and the remainder of the calories as fat (FAT:CHO + PRO ratio 4:1) [77]. Nowadays, the composition of KDs vary widely in the literature, with different proportions of macronutrients, energy content, and protocol duration [78].

Ketogenic diets may be usually normocaloric or with a reduced energy intake (low calorie or very low calorie ketogenic diet (VLCKD)) [14,79,80,81,82,83] but, in both cases, they should be rich in fats, adequate in protein and low in carbohydrates (i.e., less than 30 to 50 g/day or 5 to 10% of total daily energy intake) to produce ketosis. The term “Therapeutic Ketogenic Diet” is sometimes used to refer to severely restricted diets in both carbohydrate and protein (i.e., less than 5% of percentage of total daily energy intake and less than 20 g of CHO daily), which is typically used in the treatment of epilepsy and cancer [81].

Although there is no official classification of KDs, some authors have proposed the following types according to their daily calorie intake [78,84]:

- VLCKD (very-low-calorie ketogenic diet) with less than 700 to 800 kcal/day, carbohydrate intake < 30–50 g/day, lipid intake of up to 30 to 40 g/day and protein intake of 0.8 to 1.2 g/kg of body weight per day;
- LCKD (low-calorie ketogenic diet) with at least 700 to 800 kcal/day, but less than the daily caloric requirement total energy expenditure(TEE), carbohydrate intake < 30–50 g/day, lipid intake > 30 to 40 g/day;
- ICKD (isocaloric ketogenic diet) with caloric intake in line with the daily TEE requirement, carbohydrate intake < 30–50 g/day, lipid intake > 70–80% of daily calorie intake.

It is worth noting that a KD mandatorily restricts digestible carbohydrates, i.e., those which directly provide energy, such as starch, sucrose, lactose, glucose, whilst non-digestible carbohydrates (dietary fibers) may be recommended. Although there is no consensus on the ideal composition of dietary fat, KDs should be mainly composed by unsaturated fatty acids such as olive oil, sunflower, wheat germ, and rice oils, rather than saturated fatty acids [82] The intake of soy lecithin (commercially available in the granular form) and avocado is also recommended to increase dietary polyunsaturated fats [85]. Omega-3 polyunsaturated fatty acids’ supplementation are mandatory during the first phases of the VLCKD [86]. In this regard, some propositions of a “healthier” Mediterranean version of the KD have been made [86,87,88].

Forty years after falling into disuse due to new antiepileptic drugs, in the late 1990s the KD experienced a re-emergence in the treatment of epilepsy [89] and its interest expanded to other clinical conditions such as inflammation [83], metabolic syndrome and cancer [90]. As regards weight loss or insulin sensitivity several randomized controlled trials have shown significant short, medium, or long-term (24 months) [15] favorable effects of KD. Whether consumption of a KD offer benefits over other “traditional diets” for several outcomes has been the subject of constant debate, which is largely due to the different study protocols, the short duration of the studies, the poor quality of the studies and diets, the poor control of blood or urinary ketones, and inadequate diet adherence [81,91] Randomized trials with long-term follow-up and better control of diet adherence could provide better estimates of the overall health effects of KDs.

Given the above, the KD has been considered an interesting alternative to “killing two birds with one stone” considering its significant beneficial effects on both metabolic parameters and body composition. Nevertheless, most of the studies available in the literature do not allow a clear distinction between the effects of KDs on insulin sensitivity per se, and the effects of weight loss induced by KDs on insulin sensitivity.

### 4.1. Biochemical and Physiological Aspects of Ketogenic Diets

The metabolic state induced by a nutritional ketosis was firstly defined as “physiological ketosis” by Hans Krebs in 1966 [92]. This definition was used by this prominent biochemist to differentiate the “normal” adaptation of the organism to fasting from the pathological diabetic ketoacidosis. During starvation ketosis, the ketone bodies’ (KBs) levels of adults may rise up to 5–10 mmol·L^−1^ [93,94] whilst during nutritional ketosis levels are usually around 2–3 mmol·L^−1^ [17,95] without any pH change [96]. Physiological ketosis differs from the pathological diabetic ketosis where ketonemia can exceed 15 mmol·L^−1^ and cause blood acidosis [97]. Generally speaking, in adults a diet can be considered ketogenic when the KBs level is higher than 0.5 mmol/L [98]. Physiological ketosis is an evolutionary metabolic adaptation to face periods of undernutrition due to low food supplies [99,100].

Ketogenesis—the formation of ketone bodies (KBs)—occurs mainly at hepatocyte mitochondrial level when there is an “overflow” of fatty acids with a contemporary reduction in available glucose. KBs then leave the liver, pass in the bloodstream, and reach different tissues (mainly the brain, kidney, and skeletal muscle) to be concerted in acetyl-CoA and used as source of energy (for more detail, see Box 1).

Box 1 **Biochemical pathways of ketosis.**

When there is a condition of low glucose availability, together with low insulin, the flux of fatty acids from body’s fat depots increases. Fatty acids from the adipose tissue are oxidized in liver to produce acetyl-CoA, which, in turn, supplies the tri-carboxylic acid cycle (TCA) for generation of ATP. However, under the drastic reduction of nutritional carbohydrates (such as during a KD) and decline in glucose/glycogen reserves, the TCA cannot manage the increased flux of acetyl-CoA due to the in-creased lipolysis (mainly due to the lower level of insulin) [101]. At the same time the reduction of available glucose causes a shortage of oxaloacetate. For the functioning of the Krebs cycle oxaloacetate is necessary for the condensation reaction between it and for the thioester acetyl coenzyme A to form citrate. At normal body temperature oxaloacetate is unstable and it cannot be accumulated and stored, thus, for the functioning of the TCA, it is necessary to replenish oxaloacetate by an anaplerotic reaction involving the conversion of glucose to pyruvate to oxaloacetate (catalysed by pyruvate carboxylase). There are also other minor anaplerotic reactions which can create oxaloacetate involving glucogenic amino acids [101]. Then, the increased amount of fatty acids and the shortage of oxaloacetate (together with other regulators) [102] stimulate ketogenesis in the liver with the synthesis of ketone bodies (KBs) from Acetyl-CoAs: β-hydroxybutyrate (βHB), acetoacetic acid (ACA) and acetone [103]. The process of ketogenesis proceeds from the condensation of two acetyl CoA with the production of an acetoacetylCoa, then the acetoacetylCoa becomes 3HMGCoA through the addition of a third acetyl-Co. The HMG-CoA lyase catalyzes the passage from HMG-CoA to acetoacetate plus acetyl-CoA; the acetoacetate (AcAc, the first ketone body) is then reduced by the NADH-dependent β-hydroxybutyrate dehydrogenase to the other ketone body, the β-Hydroxybutyrate (βHB) and, by a spontaneous decarboxylation, to acetone, the third, volatile ketone [104,105,106,107,108,109]. Of the 3 named KBs, the main circulatory one is: βHB, to a minor extent, AcAc, whilst acetone is highly volatile and thus, eliminated by breath. In tissues (mainly brain, kidney, heart, and, in a less ex-tent, skeletal muscle), βHB is transformed in AcAc. Next, AcAc is activated by succinyl-CoA:3-ketoacid-coenzyme A transferase (SCOT) in AcAc-CoA to subsequently form two molecules of Acetyl-CoA by a mitochondrial thiolase. The Acetyl-CoA, at this point, may be utilized by the tissues. This mechanism allows mammals to overcome the problem posed by the blood brain barrier (BBB) which does not permit the passage of the FFA into brain. Consequently, during glucose shortage, it serves as an energy substrate for brain cells. In the brain (through a specific transporter): βHB provides the necessary Acetyl-CoA for the TCA. Interestingly, the liver, the main producer of KBs cannot use them due to the lack of the necessary enzyme SCOT [105,106,107,108,109].

From an endocrine point of view, the regulation of ketogenesis and ketolysis is influenced by several factors, such as insulin, glucagon, cortisol, catecholamines and growth hormone levels [110]. After a meal, the release of insulin promotes the activation of acetyl-CoA carboxylase, increasing the levels of malonyl-CoA. This metabolite is the precursor of fatty acid synthesis but also inhibits the activity of carnitine-palmitoyl transferase 1 and, consequently, the oxidation of fatty acids. Under these circum-stances, ketogenesis does not occur due to the lack of mitochondrial acetyl-CoA production from the oxidation of fatty acids [108,111]. During KD, reduction in malonyl-CoA also stimulates AMPK [112] while glucagon and epinephrine contribute to in-creased phosphorylation and inactivation of acetyl-CoA carboxylase activity. Thus, the activity of carnitine-palmitoyl transferase 1 is unblocked and the oxidation of fatty acids occurs, thus producing a high amount of mitochondrial acetyl-CoA, which can be used as a precursor of ketone bodies. Moreover, the reduced insulin concentration inhibits [73] HMG-CoA reductase, then reducing cholesterol production and increasing ketogenesis [113].

### 4.2. Effects of Ketogenic Diets on Weight Loss

Although the negative energy balance (energy intake < energy expenditure) plays a pivotal role in weight loss, this simple model must be viewed with caution in obesity treatment programs since obesity pathogenesis involves a complex mixture of molecular, genetic, developmental, behavioral, and environmental factors [114]. The problem with the straightforward model “calories in and calories out” in weight loss programs is the difficulty of avoiding a dysfunction in the homeostatic energy system that hinders long-term weight loss [115]. With weight reduction, hunger increases and energy expenditure (EE) decreases, both physiological adaptations that tend to bring back lost body weight [115,116,117].

In recent years, the interest in the effects of a KD on weight loss has raised new peaks both in the general media and in scientific publications. There is general consensus (based also on meta-analysis) on the positive effects of KDs on weight loss, at least in the short–medium term [91,118,119,120] and in an ecological setting. The question that remains open is about the mechanisms involved in these results [79]. The suggested mechanisms are:

(1) A spontaneous reduction in caloric intake in an environmental setting rather than metabolic ward [121,122] due:
  a. To a reduction in appetite due to higher satiety effect of proteins [123];
  b. To some effects on appetite control hormones [124];
  c. To the possible direct appetite suppressant action of the ketones bodies [125];
  d. Or to the combination of of all three factors.
(2) A small, but environmentally significant increase in resting energy expenditure (REE) [126];
(3) An increase in the rate of lipolysis (greater percentage of energy derived from fat) [127].

Some studies showed that in an isocaloric diet, proteins are more satiating than either carbohydrates or fats [128,129]; for this reason it has been suggested that the higher protein intake in KDs may have a role in reduction in energy intake [123,130]. One study in which the carbohydrate percentage was kept at 50%, whilst the protein intake was modified from 15% to 30%, demonstrated the positive effect of protein on satiety, probably through a central leptin sensitivity mechanism [131]. Another study showed that a high-protein, low-carbohydrate ICKD was able to reduce appetite and thus reduce food intake in a significant manner compared to high-protein, medium-carbohydrate non-KDs [132], with a decrease in energy intake of about 294 kcal/d. These findings were not confirmed by Hall and colleagues [133], who compared the effects of an isocaloric animal-based KD vs. a low-fat, high carbohydrate plant-based diet on *ad libitum* energy intake in a study completed in a metabolic ward (i.e., in extremely controlled conditions but not comparable to free living). The KD diet resulted in a greater energy intake despite consumption of more dietary protein than the low-fat diet. Interestingly, energy intake during the KD diet was significantly decreased during the second week compared to the first week, coinciding with the increase in βHB blood levels.

It is well known that there is a complex network of hormones and metabolites that influence satiety and hunger. Hormones, such as leptin (from adipose tissue) and insulin, are related to the amount and quality of adipose tissue. These hormones pass the blood–brain barrier and stimulate specific receptors mainly in the hypothalamic areas rich in axons from the arcuate nucleus (ARC), which has greater concentrations of leptin and insulin receptors than any other hypothalamic site [134]. The ARC plays a double role in appetite, exerting both orexigenic and anorexigenic effects, influenced not only by leptin and insulin, but also by gut hormones (ghrelin, PYY, etc.). Diet may influence the basal level of these food control-related hormones but also their response to a meal. Indeed, Sumithran and colleagues demonstrated the long-term (one year) persistence of changes in some peripheral hormones involved in food control after a very low calorie diet [63,135]. They measured a decrease in anorexigenic hormones: leptin, peptide YY, cholecystokinin, insulin, and pancreatic peptide and an increase in the orexigenic ghrelin. They found that hunger consistently remained elevated one year after diet cessation. The same group demonstrated that, in a similar experimental setting, the KD was able to suppress ghrelin increase induced by low energy [124], results that were also previously shown by another group [136]. Another important hormone related to food control and influenced by KDs is adiponectin. During the first days of fasting or KD, there is a rise of adiponectin concentration [59,137]. Adiponectin activates neurons expressing pro-opiomelanocortin (POMC), leading to a decrease in food intake. At the same time, adiponectin activates neurons producing neuropeptide Y (NPY) and agouti-related peptide, which are known to cause an increase in food intake. Interestingly the appetite control by hypothalamic arcuate the nucleus seems to be related to different nutritional states and plasma glucose concentration, as adiponectin at high glucose concentration inhibits POMC neurons and increases food intake; meanwhile at low glucose levels it exerted opposite effects suggesting an appetite role related to nutritional status [138].

Although there is no consensus about the minimum level of BHB required to reduce appetite (varying from 0.3 to 1.48 mmol/L) [98], the direct anorexigenic effect of KBs seems to be a plausible explanation linking KDs to weight loss. In this regard, Deemer and colleagues [139] wrote, in their narrative review, that appetite suppression during a ketogenic diet only lasts for as long as participants are in ketosis which infers a direct effect of KBs on appetite. This observation is confirmed by Stubbs’ data showing the direct relationship between ketones’ concentration and appetite suppression and fullness, using a ketone ester (KE) drink [140]. In this study, appetite suppression observed following KE drinks was attributed to lower levels of ghrelin. These data have been recently confirmed by our group using a ketogenic diet in which low levels of glucose were also directly related to appetite suppression [98]. The mechanism underlying the anti-hunger effect of ketone bodies has not yet been clarified and it is still unclear whether they act directly on the brain or are mediated by other molecules such as ghrelin, leptin, NPY, PYY, etc. [141] but it is clear that the appetite suppressor effect is related to the reaching of the minimum level of ketonemia. It is worth underlining that there is still a lively debate about the effects of KDs on weight loss. One of the problems may be that hunger/satiety control and other physiological adaptations to lower carbohydrate intake (e.g., hormonal responses and energy expenditure) are dependent on the length of the diet protocol, underscoring the need for longer trials to understand chronic nutrient effects on weight loss [142].

The need for longer trials on KDs is related also to the changes in REE or TEE induced by a KD. In an elegant controlled feeding trial involving overweight adults randomized to high (59%), moderate (40%), or low (20%) carbohydrate diets over 20 weeks, Ebbeling and coworkers [143] found a greater total EE in the low-carb diet group compared with the group following energy and lipid-matched but higher carbohydrate diet. The increase in TEE was about 278 kcal/day or 50 to 70 kcal/day for every 10% decrease in the contribution of carbohydrate to total energy intake during weight loss maintenance phase (i.e., under a normocaloric diet). Interestingly, the difference in total EE between diets was not attributable to REE or physical activity level, but mainly to the decreased ghrelin levels which have been reported to lower EE. Ludwig and collaborators [142] in a recent metanalysis of controlled feeding studies confirmed the greater effect of lower versus higher-carbohydrate diets on total EE. However, the advantage of low-carb diets on EE (+135.4 kcal/day) was observed only in medium–long term studies (>2.5 weeks) whereas short trials < 2.5 weeks induced a modest (and probably transient) reduction of about 50.0 kcal/day in total EE.

However, other research such as the study of Hall and collaborators has not supported the hypothesis of a significant difference in the amount of energy expenditure following a ICKD [122]. In the aforementioned study, authors investigated changes in energy expenditure, respiratory quotient (RQ), and body composition in participants consuming a high-carbohydrate baseline diet for 4 weeks followed by 4 weeks of an isocaloric KD with the same amount of protein. The results showed that large isocaloric changes in the proportion of dietary carbohydrates to fat transiently increase energy expenditure by only ~100 kcal/d after adjusting for body mass and composition. The authors also suggested that body mass and body composition adjustments probably tend to overestimate the energy expenditure changes because during a KD much of the body mass loss should be attributed to fluid loss rather than a change in metabolically active tissues. Another study by Hall and collaborators showed a trend (although not significant) for a greater degree of negative energy balance during a fat-reducing diet compared to an isocaloric carbohydrate-reducing diet [144]. These conflicting data from different studies suggest that the metabolic advantage measured during a KD may vary from small to medium, and that many variables should be considered in the math such as the energy losses from ketones and fat in breath, urine and stool [145] and also to, small, but environmentally important (≈ 50 kcal/d) [146] increase in spontaneous physical activity [144]. The effect of a KD on REE in different metabolic conditions, merits further discussion: if the above mentioned studies showed an increase in REE (from little/non-significant to greater/significant) in patients with obesity, there are data showing no changes in REE after 60 to 90 days of a very VLCKD [147], suggesting that this preservation may be attributed to the preservation of lean mass rather than to increased sympathetic tone (as thyroid hormones, catecholamines, and leptin were reduced). It is noteworthy that fat free mass (FFM) explained more than 40% of the variability in REE and despite the considerable weight loss induced by the energy restricted diet, there was a positive nitrogen balance throughout the intervention [147]. Other data confirmed the maintenance of LBM and no reduction in REE [148,149,150]. From a clinical point of view, the preservation of muscle mass is essential to avoid sarcopenic obesity which represents a double negative impact on the cardiometabolic health of patients [151]. Indeed, skeletal muscle constitutes 40% of total weight and plays many fundamental metabolic roles. KDs seem to preserve skeletal muscle mass even though the gluconeogenesis process, mandatory in producing glucose, utilizes amino acids as the source of glucose. Indeed, after a few days of a KD, the contribution of amino acids becomes less important as glucose is substituted by KBs as the energy source and some glucose also comes from the glycerol of triacylglycerols [152].

KDs improve fat oxidation and therefore lower the RQ [144,153], suggesting the increase in fat metabolism for energy use as one of the most important mechanisms of the KD on fat loss. It is important to underline, however, that the lower RQ reflects an increased percentage of energy derived from fat oxidation rather than an increase in energy expenditure [79]. However, it is important to consider that even though the weight loss induced by KDs is mainly from fat mass (~85%) [118,150] some degree of loss of lean body mass may happen [17]. During the first days of KD, the main contributor to weight loss is the water linked to muscle glycogen depots. The increased diuresis due to urinary excretion of ketones and sodium is responsible for the extracellular water decrease during the KD’s first week [154,155]. As mentioned before, a sparing of lean mass is expected to occur in normoprotein or high protein KD. However, reductions attributable to muscle mass around 1 kg have been observed in low calorie KDs along with a maintenance of bone mineral content [148]. Thus, considering some possible “side effects” of KD induced weight loss such as dehydration, hypocalcemia and muscle losses, recommendations on water (at least 2 L daily), magnesium, calcium, vitamin D supplementation, and protein intake (from 1 to 1.5 g/kg/day) are made [119].

In summary, many studies on humans have consistently showed that a KD may be considered more effective or just as effective as other types of “weight-loss” diets such as low fat, balanced or low calorie Mediterranean diet as summarized by recent meta-analysis and systematic reviews [118,119,120,156,157,158,159]. The weight loss effects of KDs are mainly attributed to a good control of satiety/appetite and/or a modulation of energy expenditure concomitantly with a “motivational pushing” as the faster weight loss in the beginning of treatment could increase the patient’s long-term adherence [15].

### 4.3. Effects of KDs on Insulin Sensitivity Mediated by Fat Changes

Between the raw effect of weight loss and the direct effects of ketosis on IR there is a “middle-ground” where the effects of KDs on IR may be modulated by the different effects of ketosis on fat tissue.

#### 4.3.1. KDs and Visceral vs. Subcutaneous Fat

It is well known that visceral fat affects insulin sensitivity to a greater extent compared to the subcutaneous fat. As a matter of fact, the increase in visceral lipids is associated with an increased availability of bioactive lipids, such as fatty acyl-CoAs, diacylglycerides, and ceramides that induce subclinical inflammation and insulin resistance [160] and a reduction in visceral adipose tissue can lead to an improvement in the metabolic health and thus of insulin sensitivity [161]. Data from our and other labs showed unequivocally that KDs are capable of significantly reducing the amount of visceral adipose tissue depots both in sedentary [17,162] and active/athletic subjects [95]. Moreover, at least in animal model, KDs reduce adipose tissue cells size.

#### 4.3.2. KD and Skeletal Muscle Insulin Sensitivity

Ketogenic diets act on skeletal muscle with an “exercise-type” mechanism [163]. It has been demonstrated that a KD, together with exercise, is able to improve skeletal muscle mitochondrial capacity and efficiency, optimizing fat oxidation and improving metabolic health markers [164].

#### 4.3.3. KDs and Liver Fat Depots and Insulin Sensitivity

Non-alcoholic liver fat disease (NAFLD) is strictly related to other metabolic diseases, such as type 2 diabetes mellitus, which, in turn, plays a pivotal role in the pathogenesis and progression of NAFLD [5,39,165]. Consequentially [166], in recent years a growing body of evidence has shown a rapid and marked reduction in liver fat accompanied by a marked decreases in body weight in NAFLD patients treated with KDs [19,167,168]. The decrease in hepatic triacylglycerol improves hepatic IR, reducing excessive hepatic glucose production and compensatory hyperinsulinemia. The lower levels of glucose and insulin in KDs also reduce the cholesterol biosynthesis mediated by β-Hydroxy β-methylglutaryl-CoA reductase, which is activated by insulin [169]. In regard to LDL-c, KDs promote shifts in the particle to a larger size decreasing the proportion of small, dense and more atherogenic LDL particles [170].

A recent study [19] showed that only 6 days of a LCKD were able to decrease the intrahepatic TG content of NAFLD individuals by 30%, and markedly improve insulin sensitivity which was determined by decreases in fasting serum glucose and insulin (−13% and −53%, respectively), *C*-peptide concentrations (−36%) and HOMA-IR (−57%). The reduction in hepatic TGs content despite an increase in free fatty acids concentration was attributed to an increased TGs hydrolysis mediated by lower insulin levels. Additionally, the increased TGs hydrolysis toward β-oxidation and subsequently ketogenesis was attributed to increased hepatic mitochondrial redox state. Considering the difficulty to dissecting the effect of weight loss from that of ketosis, these results should be considered with caution. Indeed, Kirk et al., reported a similar degree of weight loss and intrahepatic TG reduction in obese NAFLD patients comparing results after 11 weeks of KD vs. a control diet equivalent in calories [171]. Similarly, 2-week VLCKD (~1550 kcal/day) reduced both liver TG (by ~55%) and body weight (−4.6 kg) in obese subjects with NAFLD [172] with a greater decrease in hepatic TG content in the VLCKD group. KDs increase hepatic mitochondrial redox state in the liver in humans [19] and, in a rat model, its anti-steatogenic effects are mediated by the increased liver expression of genes involved in mitochondrial biogenesis and fatty acid oxidation (PGC-1α and Fgf21) and by the suppression of inflammatory genes (TNF-α, Nf-kb, and Il-6) [173]. Finally, the KD-induced reduction in fat in the liver seems to improve insulin sensitivity through the improvement of mitochondrial efficiency [19], reduction in inflammation [173], oxidative stress [174], diacylglycerol accumulation, and kinase C activity [173]

### 4.4. Direct Effects of KDs on Insulin Sensitivity

The effect of KDs on insulin sensitivity appears to also be mediated by other factors than the weight loss itself. The restriction in CHO intake decreases intestinal absorption of monosaccharides leading to reduced postprandial glycemia, decreased insulin requirements from the pancreas, and lower insulin levels and insulin-to-glucagon ratio [175,176,177]. For example, the insulin required to metabolize a ketogenic meal is almost 10 times lesser than that necessary to metabolize a Mediterranean diet meal [178].

The effectiveness of the KD in lowering fasting blood glucose was confirmed in a recent meta-analysis including T2DM patients [177] which showed a decrease of about 1.29 mmol/L in fasting blood glucose and 1.07 in glycated hemoglobin (HbA1c). The KD also improved lipid metabolism, with significant reductions in TG (−0.72 mmol/L) and total cholesterol (−0.33 mmol/L) and significant increases in HDL-c (+0.14 mmol/L). Notably, the therapeutic effects of the KD occurred despite the duration of the intervention, which ranged from 1 to 56 weeks. Considering that this study included only single-arm trials and did not analyze the homeostasis assessment of insulin resistance test (HOMA-IR), it is difficult to make inferences with certainty about an “insulin-sensitizing” advantage of KDs in relation to other diets.

Another recent meta-analysis comparing the effects of 3, 6 and 12 months of KDs with any diet recommended for patients with type 2 diabetes [179] showed that 3 and 6 months of a KD produced a more significant reduction in HbA1c, body weight, and TG and a greater increase in HDL-c compared to the other diets, with no differences in LDL-c between groups. After 12 months, dietary differences between HbA1c and weight loss disappeared, but the TG remained significantly lower in KD while LDL-c reductions were more pronounced in the control diet group. Interestingly, in the sensitivity analysis, the effect of KDs on HbA1c levels remained significant after removing studies with significant weight loss. Moreover, the KD groups had a significantly higher number of patients who decreased their glucose-lowering medications. According to the authors, the findings reveal that the benefits of KDs are observed only over the short term. However, it was not clear whether adherence to the KD was maintained over time, which may have interfered with the results. For this reason, the evaluation of β HB levels—which can be performed using commercially available monitors—is mandatory for defining the correct dietary adherence in the studies involving KDs [180]. The ingestion of natural foods (e.g., leafy greens, cabbage, artichoke, berries, chia seeds) or supplements containing dietary fibers and more speculatively also postbiotics could be considered to improve long-term glycemic and lipid control during KDs.

In a one-year study including nondiabetic individuals, Brinkworth and collaborators [181] found similar lowering effects in fasting glucose, insulin, insulin resistance and sensitivity, and *C*-reactive protein for ICKD (CHO < 20 g/day for the first 8 week and <40 g/d for the remainder of the study) compared to an isocaloric low fat diet. Despite the same weight loss, the KD group had a greater increase in HDL-c and reduction in triglycerides. Conversely, the low-fat group showed lower levels of LDL-c. Similar findings were observed in many previous randomized controlled trials comparing the effects of low or very low carbohydrate diets to low fat diets (≤30% of the daily energy intake) on cardiovascular risk factors in subjects with overweight and obesity [182,183].

Taken together, these results have a clinical significance, as the dyslipidemia is associated with insulin resistance (as mentioned above) and diabetic complications, such as atherosclerotic lesions [177]. Notably, KDs improve the lipoprotein profile (at least over periods of approx. 1 year) independently of weight loss. The low availability of dietary CHOs decreases hepatic glycogen and *de novo* synthesis of fatty acids in hepatocytes and enhances whole-body fat catabolism, thus reducing liver and blood lipids [19,176,184]. KDs could induce the expression of fibroblast growth factors promoting hepatic clearance of TGs and insulin sensitization in skeletal muscle [185,186]. Table 1 summarizes relevant meta-analyses of the effects of KD on body composition, glycemic and lipid profile.

It is worth also discussing the different action of ketosis on the numerous pathways that may influence indirectly insulin sensitivity.

#### 4.4.1. Oxidative Stress

In addition to carbohydrate restriction and its effect of boosting fatty acid oxidation, the high βHB production induced by KDs is also likely to enhance resistance to oxidative stress. Under physiological conditions mitochondria produce reactive oxygen species (ROS) as a defensive mechanism to combat cellular stress whilst excessive ROS, in turn, are scavenged by antioxidant pathways [187]. Low levels of ROS improve insulin sensitivity by facilitating modulation by insulin receptor of stress-response kinases. By contrast, increased ROS production stimulates IR. However, the picture is more complex as ROS can exert both beneficial and harmful effects on insulin sensitivity [188]: ROS can reduce the insulin response thereby increasing the risk of developing insulin resistance whilst H_2_O_2_ enhances insulin sensitivity in vivo, and a certain level of ROS is required for normal intracellular signaling [189]. Finally, a physiological balance between ROS production and antioxidant capacity is pivotal to insulin signaling. It is known that ketosis reduces oxidative stress, even though the underlying mechanisms are not still completely explained. KBs may exert their protective effects both directly and indirectly (see Box 2).

Box 2 **Direct and indirect effects of ketone bodies on oxidative stress.**

The main direct effect is the scavenger action of βHB on hydroxyl radical (∙OH), whilst the indirect effects are complex with many nuances [104,190,191]. One suggested mechanism is the improvement in mitochondrial efficiency through the increase in the redox span between complex I and II due to the reduction in the free mitochondrial NAD+/NADH ratio and the increase in the free mitochondrial CoQ/CoQH ratio [192,193,194,195] that may reduce the production of H_2_O_2_ [192,196]. Duration of exposure may be important besides the quantity of ROS: in a rat model during a ketogenic diet, after the initial first days increase in oxidative stress (H_2_O_2_) there was an activation of the nuclear factor erythroid 2-related factor 2 (Nrf2) a well-known controller of antioxidant genes such as the glutamate cysteine ligase that regulate the production of reduced glutathione (GSH) [197] the superoxide dismutase (SOD), the catalase, haem oxygenase (HO-1), and NAD(P)H:quinone oxidoreductase 1 (Nqo1) [198,199]. This hormetic typical biphasic response may help to explain some contradictory results found in the scientific literature about the effects of βHB on oxidative stress. Another mechanism involved in ROS control is the NAD+-dependent sirtuin deacetylases 3 (SIRT3) pathway. Sirtuins (Sirt1-Sirt7 in mammals) are a highly conserved protein family, in particular Sirt3, located within mitochondria, is a member of class III histone deacetylases enzymes (HDACs). Indeed, Sirt3 activates FoXo3a through its deacetylation, and promotes the activation of antioxidant genes: SOD2 [200], catalase [200], and PGC-1α [201]. βHB also directly activates PGC-1α in co-activation with FoXO1 [202]; in turn, PGC-1α increases the levels of SOD2 and catalase [203]. Sirt3 also directly activates SOD2 by deacetylation [204].

#### 4.4.2. G-Protein-Coupled Receptors (GPCRs)

Another pathway that is involved in the effects of KDs on insulin sensitivity is that of the G-protein-coupled receptors (GPCRs), the hydroxycarboxylic acid 2 HCA2/GPR109a is a GPCR that has a role in the inflammation pathway and in lipolysis. Indeed, the activation of HCA2 exerts an antilipolytic activity in different adipose tissues [205,206] representing a negative feedback for high levels of FFA that leads to a reduced glucose level [207,208]. A high level of βHB activates HCA2, explaining the reduced concentration of plasma FFAs and glucose during βHB infusion even during constant insulin level [209]. The HCA2 activation by βHB stimulates the secretion of adiponectin [210] that would lead to an enhanced AMPK activity and consequent improvements in insulin sensitivity, improves hepatic liver TGs content and, in general, insulin sensitivity [211,212,213].

#### 4.4.3. Inflammation

Many mechanisms are supposed to be involved in the anti-inflammatory effects of βHB. The main pathway is the one involving the NLRP3 (NOD-, LRR- and pyrin domain-containing protein 3) [104]. The pattern recognition receptor TLR4 (toll like receptor 4) is then activated by damage-associated molecular patterns (DAMPs) after it recognizes them. Once activated, TLR4 induces the production of NF-kB that, in turn, activates NLRP3 promoting the formation of the NLRP3 inflammasome complex that starts the inflammatory cytokines’ flux [214]. The βHB anti-inflammatory effect acts by blocking of the TLR4-mediated priming, and of the assembly of the NLRP3 inflammasome complex [215] through an inhibition of K^+^ efflux [83].

#### 4.4.4. Sirtuin Mediated Signals

Sirtuin 3 (Sirt3), is member of class II histone deacetylases, and it mediates many of the positive effects of βHB, mainly through the deacetylation of FoXo3a that promotes the activation of genes such as SOD2 [200], catalase [200], and PGC-1α [201]. Sirt3 also deacetylates IDH2 that subsequentially increases the GSH/GSSG ratio [216].

#### 4.4.5. Mitochondrial Efficiency

Ketosis may improve the mitochondrial efficiency, through some mechanisms, which were previously mentioned (see Section 4.4.1). The adaptations that enhance resistance to oxidative stress are known as mitohormesis [217]. Since many diseases—including obesity and diabetes—are associated with mitochondrial impairments, mitohormesis has been considered a target for their prevention and treatment [106,190,217,218,219,220].

#### 4.4.6. The Microbiome Connection

A prominent area of interest involves the influence of KDs on the gut microbiota and more specifically, its impacts on the etiology and treatment of many diseases [221]. Despite the very sparse literature, a link between the gut microbiome and insulin sensitivity is claimed [184,222]. Gut microbes produce SCFAs such as butyrate, propionate, and acetate which are the main energy sources for human colonocytes and act as the signaling molecules between the gut microbiota [221]. Although the exact mechanisms are still speculative, the beneficial effects of a correct KD (rich in dietary fibers and microbiota-accessible carbohydrates—MACs) on insulin sensitivity may be connected with its ability to increase *Akkermansia Muciniphila* [223]—a mucin degrading bacterium (even though many authors suggest only an association and not a causation [224])—and other specific SCFAs-producing bacteria such as *Bifidobacterium pseudocatenulatum* [225].

As regards glucose metabolism, SCFAs stimulate the secretion of two gut hormones, peptide YY (PYY) and glucagon-like peptide-1 (GLP-1) [226]. While PYY increases glucose uptake in muscle and adipose tissue, GLP-1 increases insulin and decreases glucagon production in the pancreas [134].

It is reasonable to assume that the inclusion of non-starchy vegetables in a KD helps to guarantee not only an adequate micronutrient status but also fibers which are used as substrate for the gut microbiota produce SCFAs [217]. The inclusion of supplements and/or food sources of prebiotics (e.g., onion, garlic, asparagus etc.) and low-carb probiotics (e.g., yogurt, pickles, kefir) is as well of great importance for gut microbiota’s health during the KD [221]. Moreover, vegetable protein sources in place of animal products could prevent proteolytic fermentation and reduce the negative effects of high protein KD on microbiome [227].

All of the above discussed pathways are related to an improvement in insulin sensitivity and thus, can help to explain the positive direct effects of the ketogenic diet on insulin sensitivity [228,229].

**Figure 1:**
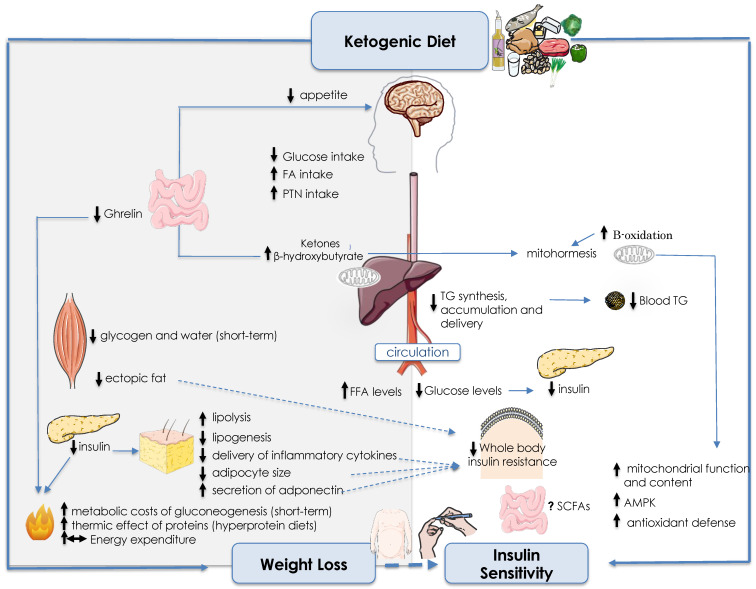
Summary of suggested mechanisms of ketogenic diets on weight loss (**left**) and insulin sensitivity (**right**). The severe restriction in digestible carbohydrates’ intake reduces insulin secretion leading to decreased lipogenesis, and increased lipolysis and blood levels of ketone bodies (KBs). Ketone bodies may reduce appetite—and consequently energy intake—directly (via mechanisms not yet elucidated) or indirectly (via hormones). The increased rate of lipolysis contributes to weight loss by reducing fat deposits (in many tissues) and adipocyte size, leading to a decreased delivery of inflammatory cytokines and increased secretion of adiponectin, that, in turn, improve whole body insulin signaling. The low availability of dietary digestible carbohydrates also decreases hepatic glycogen, de novo synthesis, and delivery of triglycerides (TG) from hepatocytes, improving liver insulin sensitivity and blood lipoprotein profile independently of weight loss. Additionally, the increased TG hydrolysis toward β-oxidation and subsequently ketogenesis enhance mitochondrial function and resistance to oxidative stress (known as mitohormesis), improving insulin signaling. There is conflicting evidence regarding the effects of KDs on energy expenditure and short-chain fatty acids (SCFA) production by gut microbiota, which is dependent on many aspects, such as the content of dietary protein, fiber, probiotics, etc. Dotted lines indicate the mediated effects of weight loss on insulin sensitivity. Abbreviations: Fatty acids (FA); Protein (PTN); Free fatty acids (FFAs); Adenosine monophosphate-activated protein kinase (AMPK).

Figure 1 summarizes the proposed mechanisms of action of ketogenic Diet on weight loss and insulin sensitivity.

## 5. Safety of KDs

It is worth mentioning that despite KDs having been used safely for at least a century to treat drug-resistant epilepsy in children, some concerns involve its risks in patients with diabetes. KDs are not indicated in some clinical conditions including pregnancy and breastfeeding, chronic renal disease, use of sodium/glucose cotransporter 2 (SGLT2) inhibitors (risk of euglycemic diabetic ketoacidosis) and unstable angina [90,119]. Moreover, KDs in diabetic patients should be followed under medical and nutritionist supervision, with regular assessment of glucose and lipid profile.

## 6. Conclusions

Ketogenic diets improve insulin sensitivity through their irrefutable effects on fat and weight loss. Besides weight loss, KD produce direct insulin-sensitizing effects which are mostly due to the capacity of its restricted-digestible carbohydrates content to lower blood glucose and insulin levels. In addition, ketone bodies appear to be able to influence insulin signaling directly. Despite the promising role of KD on T2DM prevention, future studies should address its long-term efficacy and safety in diabetic individuals. The focus should be on the particular features of KDs, e.g., their addition of fiber through natural foods, supplements, MACs, probiotics and postbiotics in contrast to other particular features of carbohydrate restriction diets.

## Figures and Tables

**Table 1:** Summary of human meta-analysis about the effects of ketogenic diets on body composition, glycemic, and lipid profile in subjects with overweight/obesity and/or diabetes.

Population Characteristics (N, Age, Sex)	Study DESIGN	Intervention	Control Diet	Duration	Body Composition Outcomes	Glycemic and Lipid Profile	Reference
N = 1415; >18 years; M and F; BMI > 27.5 kg/m^2^	Meta-analysis of RCT	VLCKD (<50 g CHO/day or 10% of DE)	LFD (<30% energy from fat	≥12 months	↓ Greater weight loss in VLCKD groups WMD −0·91 (95% CI −1.65 to −0.17) kg	↔ No differences between dietary groups for GLU, insulin, HbA1c, and CRP levels ↓ Greater TG reduction in VLCKD groups −0.18 mmol/L (WMD; 95% CI −0.27 to −0.08) ↑ Greater increase in HDL-C in VLCKD groups 0.09 mmol/L (WMD; 95 % CI 0.06 to 0.12) ↑ Greater increase in LDL-C in VLKD groups 0.12 mmol/L (WMD; 95 % CI 0.04 to 0.2) ↓ Greater reduction in DBP in VLKD groups −1.43 mmHg (WMD; 95 % CI −2.49 to −0.37)	[118]
N = 835 ≥18 years; M and F; overweight or obesity.	Meta-analysis of noncontrolled, controlled, and RCT	VLCK (30 to 50 g CHO/day and DE ≤ 800 kcal; protein, 0.8–1.2 g/day for an ideal body weight)	Any other weight loss diet	3 weeks to 24 months	↓ Greater mean weight loss and FM loss in KD groups respectively −7.06 kg (95% CI −11.16 to −2.97) and −9.35 kg (95% CI −13.29 to −5.41) ↔ No differences between dietary groups for FFM	↔ No differences between dietary groups for GLU, HbA1c and LDL-c ↓ Greater reduction in HOMA-IR index in VLKD groups –1.36 (WMD; 95% CI –2.14 to –0.57) ↓ Greater reduction in cholesterol in VLKD groups –7.13 mg/dL (WMD; 95% CI –9.71 to –4.55) ↓ Greater reduction in TG in VLKD groups –29.90 mg/dL (WMD; 95% CI –42.47 to –17.32)	[119]
N = 801 M and F ≥18 years; overweight or obesity.	Meta-analysis of sentinel studies, including observational studies and RCT	VLCKD (CHO < 50 g/day and <800 kcal/day)	Other low or very low-calorie diets	3 weeks to 24 months	↔ No differences between VLCKD and very low-calorie diets for weight loss; −10.0 kg (95% CI −13.2 to −6.8) ↓ Greater mean weight loss in VLCKD groups compared to low calorie diet groups		[120]
N = 1282 M and F patients with type 2 diabetes.	Meta-analysis of observational studies and RCT	KD or low carbohydrate diets (≤50 g CHO/day or ≤20% of DE from CHO)	Other diets		↓ Greater weight loss in KD −2.67 kg (SMD; 95% CI −4.05 to −1.28 kg) ↔ No differences between KD and other diets for BMI	↓ Greater reduction in HbA1c in KD diet compared to control diets −1.45% (SMD; 95% CI −2.73 to −0.17%)	[156]
N = 567 ≥18 years; M and F patients with type 2 diabetes.	Meta-analysis of single-arm trials (pre-post studies)	VLCKD, LCF and VLCK (≤50 g CHO/day or ≤14% of DE from CHO)	No comparison groups	1 to 56 weeks	↓ body weight 8.66 kg (MC; 95% CI −11.40 to −5.92) after the intervention of KDs ↓ WC 9.17 cm (MC; 95% CI −10.67 to −7.66) after the intervention of KDs ↓ BMI −3.13 kg/m2 (MC; 95% CI −3.31 to 2.95 kg/m^2^)	↓ GLU 1.29 mmol/L (MC; 95% CI −1.78 to −0.79) after the intervention of KDs ↓ HbA1c −1.07% (MC; 95% CI −1.37 to −0.78) after the intervention of KDs ↓ TG −0.72 mmol/L (MC; 95% CI −1.01 to −0.43) after the intervention of KDs ↓ TC −0.33 mmol/L (MC; 95% CI −0.66 to −0.01) after the intervention of KDs ↓ LDL-C −0.05 mmol/L (MC; 95% CI −0.25 to 0.15) after the intervention of KDs ↑ HDL-C 0.14 mmol/L (MC; 95% CI 0.03 to 0.25) after the intervention of KDs	[177]
N = 648 ≥18 years; M and F (65% and 100% of study participants were female) patients with type 2 diabetes, overweight or obesity.	Meta-analysis of RCT	VLCK (<50 g CHO/day or <10% of DE from CHO)	Any recommended diet for type 2 diabetes	4 to 12 months	↓ Greater weight loss in VLCK diets after 3 and 6 months respectively −2.91 kg (WMD; 95% CI −4.88 to −0.95) and −2.84 kg (WMD; 95% CI −5.29 to −0.39) ↔ No differences between dietary groups for weight loss after 12 months	↓ Greater reduction in HbA1c in VLCK diets after 3 and 6 months respectively −6.7 mmol/mol (WMD; 95% CI −9.0 to −4.4) and −6.3 mmol/mol (WMD; 95% CI −9.3 to −3.5) ↔ No differences between dietary groups for HbA1c after 12 months ↓ Greater reduction in TG in VLCK diets after 6 and 12 months respectively −18.36 mg/dL (WMD; 95% CI −24.24 to −12.49) and −24.10 mg/dL (WMD; 95% CI −33.93 to −14.27) ↔ No differences between dietary groups for LDL-C after 3 and 6 months ↑ Greater increase in LDL-C in VLCKD groups after 12 months 6.35 mg/dL (WMD; 95% CI 2.02 to 10.69) ↑ Greater increase in HDL-C in VLCKD groups after 3, 6 and 12 months respectively 1.27 mg/dL (WMD; 95% CI 0.32 to 2.22); 3.01 mg/dL (WMD; 95% CI 0.41 to 5.61) and 1.88 mg/dL (WMD; 95% CI 0.37 to 3.39)	[179]
N = 322 ≥18 years; M and F; overweight or obesity.	Meta-analysis of experimental and quasi-experimental studies	KD (20–70 g CHO/day or ≤10% of a 2000 kcal/day diet)	No comparison groups or other types of diets or usual care	6 weeks to 1 year	All studies showed a decrease in weight, BMI, and BFP in participants on a KD	GLU, HbA1c, and fasting insulin, decreased in all studies All studies showed a decrease in triglycerides Conflicting results were observed between studies for HDL-C and LDL-C.	[180]
N = 447 ≥16 years M and F overweight or obesity.	Meta-analysis of RCT	ILCD (≤60 g CHO/day)	Low-fat diet	6 to 12 months	↓ Greater weight loss in isocaloric low-carbohydrate diet compared to low fat diets after 6 months −3.3 kg (WMD; 95% CI −5.3 to −1.4) ↔ No differences between dietary groups for weight loss after 12 months	Conflicting results were observed between the dietary interventions for GLU and insulin. ↑ Greater increase in HDL-C in isocaloric low-carbohydrate diets after 6 months 4.6 mg/dL (WMD; 95% CI 1.5 to 8.1) ↓ Greater reduction in TG in isocaloric low-carbohydrate diet after 6 and 12 months respectively −22.1 mg/dL (WMD; 95% CI −38.1 to −5.3) and −31 mg/dL (WMD; 95% CI −59.3 to −2.7) ↓ Greater reduction in TC and LDL-C in low fat diets after 6 and 12 months.	[182]
N = 606 ≥16 years M and F people with pre-diabetes or type 2 diabetes	Meta-analysis of RCT	VLCK (≤50 g CHO/day)	Diet containing a carbohydrate content above 50 g/day (all comparison diets were low fat)	3 to 24 months	↔ No differences between dietary groups for weight loss, BMI, WC, FM, FFM, after 12 months.	↔ No differences between dietary groups for HbA1c, GLU, fasting insulin, HOMA-IR, TC and LDL, after 12 months. ↓ Greater reduction in TG in VLCK after 12 months −0.28 mmol/L (MC; 95% CI −0.44 to −0.11) ↑ Greater increase in HDL-C in VLCK after 12 months 0.04 mmol/L (MC; 95% CI 0.01 to 0.08)	[158]
N = 611 ≥18 years M and F people with type 2 diabetes and overweight or obesity.	Meta-analysis of RCT	KD (<50 g CHO/day)	Other diets than KDs	3 months to 2 years	↓ Greater weight loss in KD groups −5.637 kg (SMD; 95% CI −9.76 to −1.49); ↓ Greater reduction in WC in KD groups −2.32 cm (SMD; 95% CI −4.58 to −0.06); ↔ No differences between dietary groups for BMI.	↔ No differences between dietary groups for GLU, insulin, HOMA-IR, TC and LDL-C ↓ Greater reduction in HbA1c in KD groups −0.38% (SMD; 95% CI −0.61 to −0.16%) ↓ Greater reduction in TG in KD groups −0.36 mmol/L (SMD; 95% CI −0.55 to −0.18) ↑ Greater increase in HDL-C in KD groups 0.28 mmol/L (SMD; 95 % CI 0.09 to 0.46)	[159]

M: male; F: female; CT: clinical trial; RCT: randomized controlled trials; LFD: low fat diet; VLCK: very-low-carbohydrate ketogenic diet; VLCKD: very-low-calorie ketogenic diet; LCF: Low-carbohydrate high-fat diet; KD: ketogenic diet; ILCD: isocaloric low-carbohydrate diet; NA: not applicable; DE: daily energy intake; CHO: carbohydrates; weighted mean difference (WMD); standardized mean difference (SMD); MC: mean change; FM: fat mass; FFM: fat free mass; WC: waist circumference; BMI: body mass index; BFP: body fat percentage, TG: triglycerides; TC: total cholesterol; HDL-C: HDL-cholesterol; LDL-C: LDL-cholesterol; SBP: systolic blood pressure; DBP: diastolic blood pressure; GLU: blood glucose, HbA1c: glycated hemoglobin A1c, CRP: *C*-reactive protein; HOMA-IR: homeostatic Model of Assessment for Insulin Resistance; ↔ No differences between dietary groups; ↓ statistically significant decrease (*p* ≤ 0.05); ↑ statistically significant increase (*p* ≤ 0.05).

## Data Availability

Not applicable.
